# First person – José Ignacio Quesada Márquez

**DOI:** 10.1242/bio.062685

**Published:** 2026-05-26

## Abstract

First Person is a series of interviews with the first authors of a selection of papers published in Biology Open, helping researchers promote themselves alongside their papers. José Ignacio Quesada Márquez is first author on ‘ Uncoupling of nutrient sensing and cell size control by specific defects in ceramide structure’, published in BiO. José Ignacio conducted the research described in this article while an undergraduate research assistant in Rafael Lucena Hernández's lab at the University of Seville, Seville, Spain. He is now a PhD student in the lab of Francisca Martínez Real at Centro Andaluz de Biología del Desarrollo (CABD), Seville, Spain, investigating gene regulation and morphogenesis.

**Figure:**
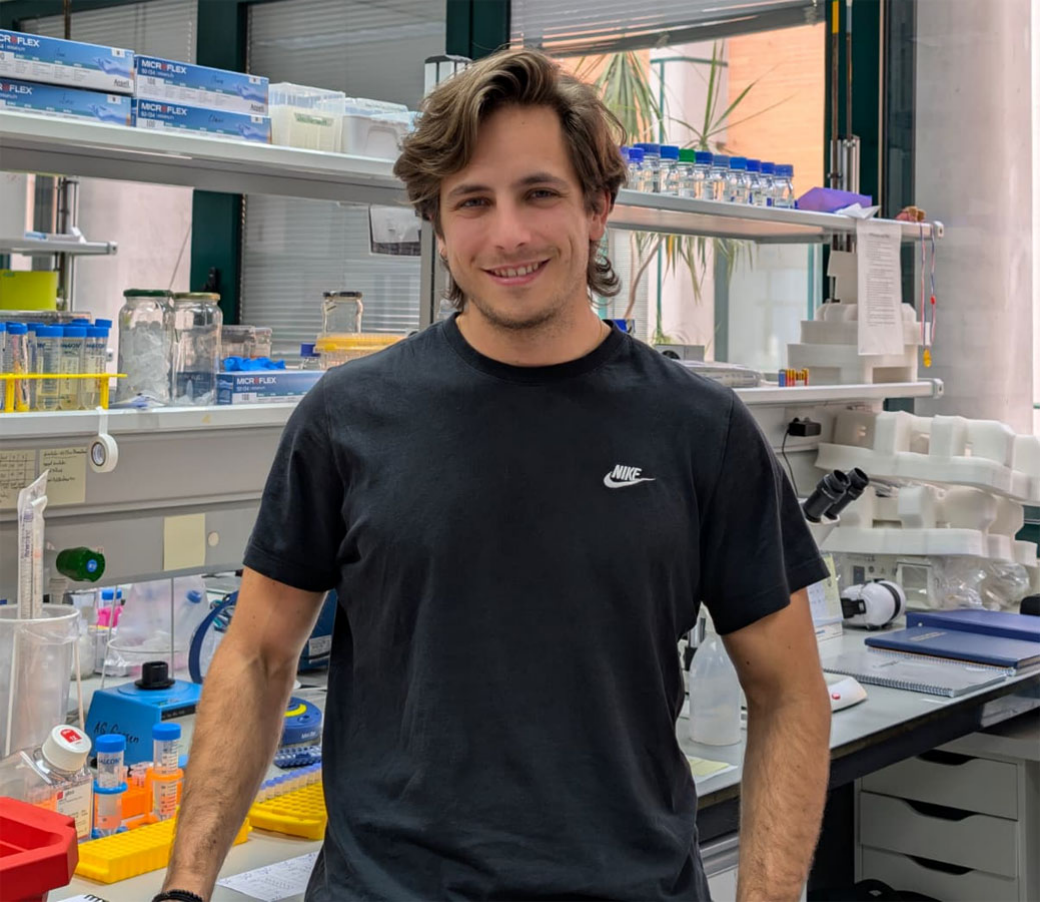
José Ignacio Quesada Márquez

**Describe your scientific journey and your current research focus**

My scientific career began when I joined the Department of Cell Biology at the Faculty of Biology of the University of Seville as an undergraduate research assistant under the supervision of Dr Rafael Lucena. During those years, I built the foundations of my career as a researcher, learning numerous techniques related to molecular biology and yeast research that later became essential to my current expertise. The work I carried out in this laboratory allowed me to complete both my Bachelor's and Master's theses, ultimately earning my degree in biology with specialization in biomedical research, physiology, and neuroscience.

From that point onward, I developed a strong interest in gene regulation, evolution, and morphogenesis, fields somewhat outside my original specialization, yet deeply fascinating to me since the beginning of my training as a biologist. I became particularly interested in understanding how a single cell can give rise to a fully developed organism with highly specialised tissues and organs. This extraordinary complexity is made possible by intricate gene regulatory networks that allow cells with the same genetic information to adopt completely different transcriptional identities and functions.

For this reason, I am currently pursuing my PhD focused on limb development in the Iberian mole, *Talpa occidentalis*, a fascinating model organism due to the remarkable morphological and physiological adaptations it has evolved to thrive in a subterranean environment.

**Who or what inspired you to become a scientist?**

My main motivation for becoming a scientist is the opportunity to explore the unknown, to answer questions that no one has asked before and to turn my curiosity into a profession. Beyond the passion and sense of responsibility I feel toward expanding the boundaries of our collective knowledge, I am also deeply attracted to the technical complexity of science. I am fascinated by the development of techniques capable of generating enormous amounts of data, allowing us to draw unique conclusions and make discoveries that would otherwise remain hidden.

**How would you explain the main finding of your paper?**

Our study shows that cells use specific lipid molecules in their membranes to sense nutrient availability and control how much they grow. We discovered that the exact size of these molecules is critical for this process to work properly. Understanding how cells regulate their size is important because this process is altered in diseases such as cancer. By uncovering new mechanisms that connect metabolism with growth control, our work provides insights with potential biomedical relevance.

**What are the potential implications of this finding for your field of research?**

These findings provide a new framework for understanding how cells coordinate metabolism, nutrient sensing, and growth. Our work suggests that membrane lipids are not simply structural components, but active regulators that help cells ‘measure’ whether conditions are appropriate for growth.

This has important implications because defects in growth control and cell size regulation are hallmarks of diseases such as cancer and metabolic disorders. By uncovering how cells precisely tune these processes, our study could help guide future research into the molecular mechanisms underlying these diseases and potentially identify new therapeutic targets.

I believe that good science is not produced in overly demanding or dehumanizing environments, but rather in laboratories where human connection, respect, and support are valued

**Figure:**
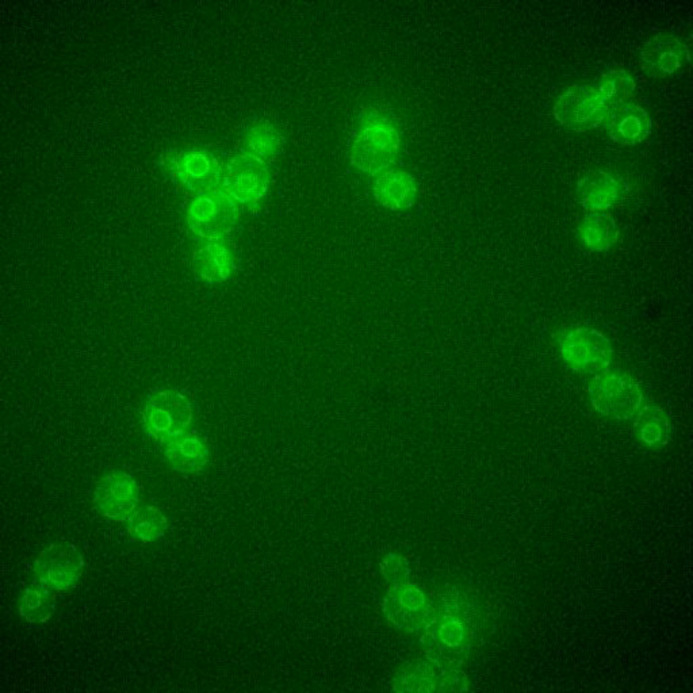
Fluorescent microscopy image of *Saccharomyces cerevisiae* cells expressing an Elo2–GFP fusion protein, showing the subcellular localisation of Elo2 in living yeast.

**Which part of this research project was the most rewarding?**

The most rewarding part of my journey has been the opportunity to learn from and work with professional and kind people who always prioritised my learning and well-being above anything else. I believe that good science is not produced in overly demanding or dehumanising environments, but rather in laboratories where human connection, respect, and support are valued. Science can be a very challenging profession that forces you to learn how to deal with frustration, but a constructive and welcoming environment can make that path far more manageable, and that is exactly what I found in Rafael Lucena's laboratory.

**What do you enjoy most about being an early-career researcher?**

What I enjoy the most is the freedom to decide how to approach problems, design my own experiments, and find the most efficient way to move forward. Although research is often a slow process, I can feel my problem-solving skills gradually improving over time, and I believe this is a valuable ability that extends far beyond science into many other aspects of life.

**What piece of advice would you give to the next generation of researchers?**

Stay curious, be open to learning outside your comfort zone, and do not be discouraged by failure. Research is built on persistence, and some of the best discoveries come from asking unconventional questions and continuing even when experiments do not go as planned.

**What's next for you?**

I am currently in the second year of my PhD, studying how changes in the regulatory landscape of the HoxD genes contribute to the morphological adaptations of mole limbs. In this project, we perform large-scale genomic modifications in mice to functionally test regulatory elements identified in the mole genome.

I find this a fascinating project, and I hope it will lead to unique and groundbreaking insights into gene regulation and morphogenesis.
